# Analysis of the differences in immune-related genes and immune cell subtypes in acute myocardial infarction

**DOI:** 10.1590/1414-431X2024e14345

**Published:** 2024-10-14

**Authors:** Zhengmei Li, Ling Kang, Ke Jiang

**Affiliations:** 1 School of Radiology, Shandong First Medical University & Shandong Academy of Medical Sciences, Tai'an, Shandong Province, China; 2 Department of Cardiology, The Second Affiliated Hospital of Shandong First Medical University, Tai'an, Shandong Province, China

**Keywords:** Acute myocardial infarction, ST-segment elevation myocardial infarction, Immune infiltration, Functional enrichment, Key genes

## Abstract

Acute myocardial infarction (AMI) continues to be a leading cause of death globally, with distinct immune cell dynamics in ST-segment elevation myocardial infarction (STEMI) and non-ST-segment elevation myocardial infarction (NSTEMI) playing a critical role in disease progression and patient outcomes. Sample data for STEMI and NSTEMI were downloaded from the Sequence Read Archive (SRA) database (https://www.ncbi.nlm.nih.gov/sra). Differences and correlations of immune infiltrating cells were assessed by CIBERSORT. Differentially expressed genes (DEGs) were identified between STEMI and NSTEMI, followed by functional analysis. Immune-related DEGs were further identified. Some immune-related DEGs were selected to perform expression verification using real-time PCR. There was a significant difference in immune cells between STEMI and NSTEMI, including activated dendritic cells, memory CD4 T cells, mast cells, and CD8 T cells. A total of 229 DEGs were identified, with functions related to inflammatory regulation and drug metabolism. A total of 21 immune-related DEGs, which may play important roles in STEMI and NSTEMI, were identified. Among the 21 immune-related DEGs, genes like *CCL18*, *NRP2*, *CXCR2*, *CXCL9*, *KIR2DL4*, *BPIFB1*, and *IL33* were significantly correlated with immune cells and had a tendency for differential expression between STEMI and NSTEMI patients. Our study reveals differences in the distribution of immune cell subsets between STEMI and NSTEMI, highlighting key immune-related genes and their association with immune cells, which may provide new insights into the treatment of AMI.

## Introduction

Acute myocardial infarction (AMI) is a disease that occurs when severe and sustained ischemia of the coronary myocardium leads to myocardial necrosis (1). Clinically, AMI is characterized by symptoms such as fever, increased white blood cell counts and serum markers of myocardial necrosis, and progressive changes in the electrocardiogram (ECG) (2). It may cause arrhythmia, shock, or heart failure (3). Changes in ECG are important means of clinical diagnosis of AMI, which can be classified into ST-segment elevation myocardial infarction (STEMI) and non-ST-segment elevation myocardial infarction (NSTEMI) according to the changes in ECG (4,5). While STEMI and NSTEMI share a common pathological foundation of coronary artery obstruction, they are different in electrocardiographic profiles, clinical manifestations, management strategies, and prognostic implications (6). It is essential to understand the difference between STEMI and NSTEMI to improve patient care and enhance survival rates.

Diverse immune cells and responses are pivotal in myocardial necrosis and recovery following AMI (7). Overwhelming inflammation or inadequate suppression of inflammatory reactions could impact myocardial healing and contribute to ventricular remodeling (8). One previous study has shown that macrophages are important regulators of the inflammatory process after AMI, with eosinophils also implicated in the pathogenesis of AMI due to their pro-inflammatory role (9). Immune infiltration analysis is a new tool for estimating immune cell subtype abundance (10). Several studies have elucidated the characteristics of infiltration of immune cells in AMI (11,12). However, the differential immune cell profiles between STEMI and NSTEMI remain unclear. In this study, we combined microarray profiles of STEMI and NSTEMI and explored the abundance and differences of 22 kinds of immune cell types using CIBERSORT algorithm. In addition, we identified immune-related differential genes (DEGs) between STEMI and NSTEMI for further analysis. Our study provides new insights into the search for treatments for AMI.

## Material and Methods

### Data set sources

The SRP053296 dataset was downloaded from the Sequence Read Archive (SRA) database (https://www.ncbi.nlm.nih.gov/sra), which contains platelet sample data from 32 patients with AMI (16 STEMI and 16 NSTEMI) and two normal controls. First, the SRA data was split by the default parameters of SRA-Toolkit software (version 3.0.0, NIH, USA). Fastp software (version 0.20.1, https://github.com/OpenGene/fastp, China) was used for quality control of fastq data. The Salmon software (version 1.3.0, https://github.com/COMBINE-lab/salmon, USA) was used for data quantification. Transcript per kilobase million (TPM) values were obtained for further analysis.

### Evaluation of immune cell infiltration in AMI

CIBERSORT, an analytical tool from Alizadeh Laboratories (https://cibersortx.stanford.edu/), was used to estimate the abundance of immune cells. Therefore, the fraction of immune cells in patients with AMI was quantified by CIBERSORT and visualized by the “ggplot2”, “pheatmap”, “vioplot”, and “ggcorrplot” packages in R software.

### Identification and functional analysis of DEGs

The “limma” package in R was employed to identify DEGs between STEMI samples and NSTEMI samples with |log_2_ fold change (FC)|>1 and P<0.05. The “pheatmap” and “ggplot2” packages were used to visualize DEGs, which were presented in heatmaps and volcano plots. In addition, to further explore the biological functions of DEGs, Gene Ontology (GO) and Kyoto Encyclopedia of Genes and Genomes (KEGG) enrichment analyses were conducted through the Database for Annotation, Visualization, and Integrated Discovery (DAVID, https://david.ncifcrf.gov/tools.jsp).

### Screening of immune-related DEGs

We acquired immune-related genes from the Immport database (https://www.immport.org/shared/home) and identified immune-related DEGs by overlapping them with the DEGs. The online database Search Tool for the Retrieval of Interacting Genes was used to predict PPI networks. In the PPI network, some genes were chosen according to a confidence score >0.15. The correlation between immune-related DEGs and immune cells was calculated by the “Pearson” method.

### Analysis of key genes using real-time PCR (RT-PCR)

The mRNA expression of selected immune-related DEGs in AMI samples was explored by RT-PCR. Nine STEMI samples and 6 NSTEMI samples were collected from 15 patients. Detailed clinical information, mainly including age, gender, BMI, alcohol, smoking, and family history, is reported in Supplementary Table S1. In addition, low high-density lipoprotein (HDL), high low-density lipoprotein (LDL), and total cholesterol (TC) were used to evaluate the risk of cardiovascular disease, and elevated cardiac troponin indicates myocardial damage or necrosis. The study was approved by the Ethics Committee of the Second Affiliated Hospital of Shandong First Medical University (2021-019) and written informed consent was obtained from every participant.

The inclusion criteria for patients with AMI were: 1) patients met the diagnostic criteria of STEMI or NSTEMI; 2) patients had AMI for the first time and did not take any special medication; and 3) patients' clinical data and laboratory examination data were complete. The exclusion criteria were: 1) patients had a history of cardiovascular and cerebrovascular diseases; 2) patients presented with severe infectious, systemic inflammatory, autoimmune, and malignant conditions; 3) patients with family history of cardiovascular diseases; 4) recurrent patients; 5) and patients who had incomplete clinical data. There was no significant difference in age, gender, and BMI between the STEMI group and the NSTEMI group.

Total RNA was isolated using the RNAliquid ultra speed whole blood (liquid sample) total RNA extraction kit (Beijing Huitian Dongfang, China). mRNA was synthesized by reverse transcription using the FastKing cDNA first-strand synthesis kit (TIANGEN, China). RT-PCR was employed to identify alterations in gene expression. Relative gene expression values were determined using the 2^-△△ct^ method. *ACTB* and *GAPDH* were used as internal reference genes. Primers are shown in the Table 1.

**Table 1:** Primer sequences in the RT-PCR.

Primer	Primer sequence (5' to 3')
GAPDH	F: GGAGCGAGATCCCTCCAAAAT
	R: GGCTGTTGTCATACTTCTCATGG
ACTB	F: CATGTACGTTGCTATCCAGGC
	R: CTCCTTAATGTCACGCACGAT
NRP2	F: CCAACGGGACCATCGAATCTC
	R:CCAGCCAATCGTACTTGCAGT
S100P	F: TGCTGATGGAGAAGGAGCTAC
	R: GCAGCCACGAACACTATGAAC
KIR2DL4	F: AGTGACCCACTGCCTGTTTCT
	R: CCAACTGTGCGTATGTCACCT
BPIFB1	F: TTCCCTCAGCATTGACCGTC
	R: GATGAGGCTGAACGGGATGT
CCL18	F: TGTGAGTTTCCAAGCCCCAG
	R: GGAGGTATAGACGAGGCAGC
CXCR2	F: CCTGTCTTACTTTTCCGAAGGAC
	R: TTGCTGTATTGTTGCCCATGT
IL33	F: GGAGTGCTTTGCCTTTGGT
	R: CCTGGTCTGGCAGTGGTTT
CXCL9	F: GAAATTGAGCTGGACCTCACC
	R: AGGCTCTGAAAGCCACACACT

GAPDH and ACTB: internal references.

### Statistical analysis

All statistical analyses were performed using R version 4.0.5. The “limma” package was used to identify DEGs, and the DEGs were visualized by volcano map and heatmap. The heatmap of 22 immune cell types was displayed using the heatmap package. The fraction of immune cells was calculated using the “CIBERSORT” algorithm, and the “ggcorrplot” package was used to plot the correlation heatmap. Bar and violin plots were plotted using the “ggplot2” package to show differences in the abundance of immune cells between the STEMI group and NSTEMI group. The differences in cell composition between the two groups were compared using the Wilcoxon test. Pearson correlation was used to calculate the correlation between genes and immune cells. All analyses were considered statistically significant with P<0.05.

## Results

### Analysis of infiltrating immune cells

**Figure 1:**
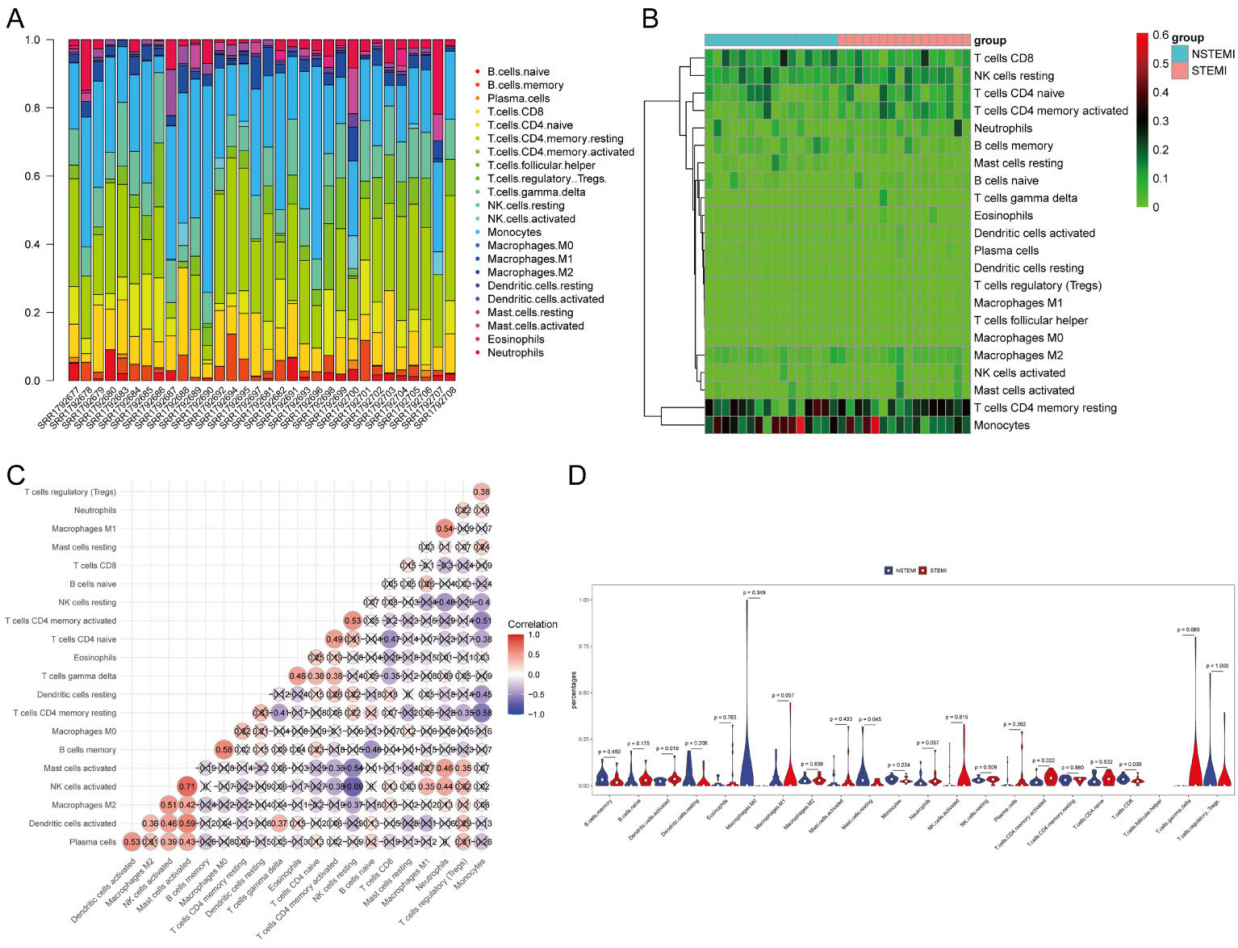
Analysis of infiltrating immune cells. **A**, Percentage of different immune cells in ST-segment elevation myocardial infarction (STEMI) and non-ST-segment elevation myocardial infarction (NSTEMI) samples. **B**, Heatmap of immune cell types in STEMI and NSTEMI. Blue represents NSTEMI and red represents STEMI. **C**, Correlation heatmap of immune cell types in STEMI and NSTEMI. Red and blue represent positive relationship and negative relationship, respectively. “X” represents P>0.05. **D**, Bar plot of difference in immune cell infiltration between STEMI and NSTEMI. P<0.05 was considered statistically significant (Wilcoxon test).

An exploration was conducted on the differences in immune cell abundance between STEMI and NSTEMI samples (Figure 1A and B). CIBERSORT results revealed a larger proportion of resting CD4 memory T cells and monocytes in STEMI and NSTEMI samples. Furthermore, the results of the correlation analysis revealed significant associations among specific subpopulations of immune cells, including a positive correlation between activated NK cells and mast cells (r=0.71, P<0.05), as well as a negative correlation between activated NK cells and resting NK cells (r=-0.69, P<0.05) (Figure 1C). Compared with NSTEMI, the infiltration level of activated dendritic cells and activated CD4 memory T cells was significantly increased in STEMI. The proportion of resting mast cells and CD8 T cells was significantly reduced in STEMI (Figure 1D).

### Identification and functional enrichment analysis of DEGs

**Figure 2:**
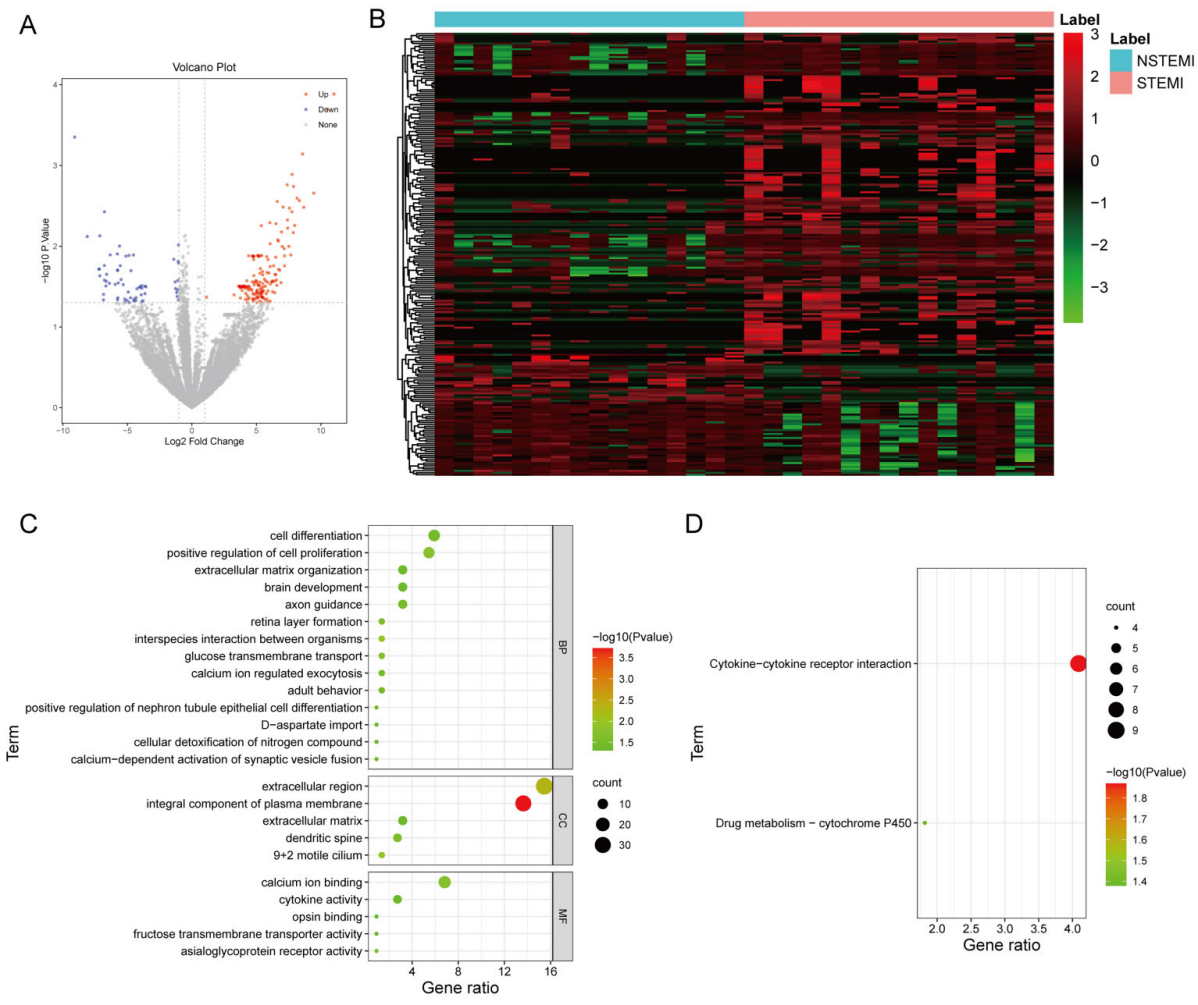
Identification and functional enrichment analysis of differentially expressed genes (DEGs). **A**, Volcano plot and (**B**) heat map show 229 DEGs between ST-segment elevation myocardial infarction (STEMI) and non-ST-segment elevation myocardial infarction (NSTEMI) samples. **C**, Gene Ontology (GO) and (**D**) Kyoto Encyclopedia of Genes and Genomes (KEGG) enrichment analysis according to 229 DEGs. BP: biological process; CC: cellular component; MF: molecular function.

A total of 229 DEGs were identified between NSTEMI and STEMI samples, including 166 up- and 63 down-regulated genes (Figure 2A and B). Furthermore, GO results indicated that these DEGs were mainly enriched in various biological processes (BP) such as cell differentiation, cell proliferation regulation, extracellular matrix organization, and brain development (Figure 2C). The mainly enriched cellular components (CC) included the extracellular region and integral component of plasma membrane. The mainly enriched molecular functions (MF) included calcium ion binding. KEGG results revealed that these DEGs were related to cytokine-cytokine receptor interaction and drug metabolism-cytochrome P450 (Figure 2D).

### Screening of immune-related DEGs

**Figure 3:**
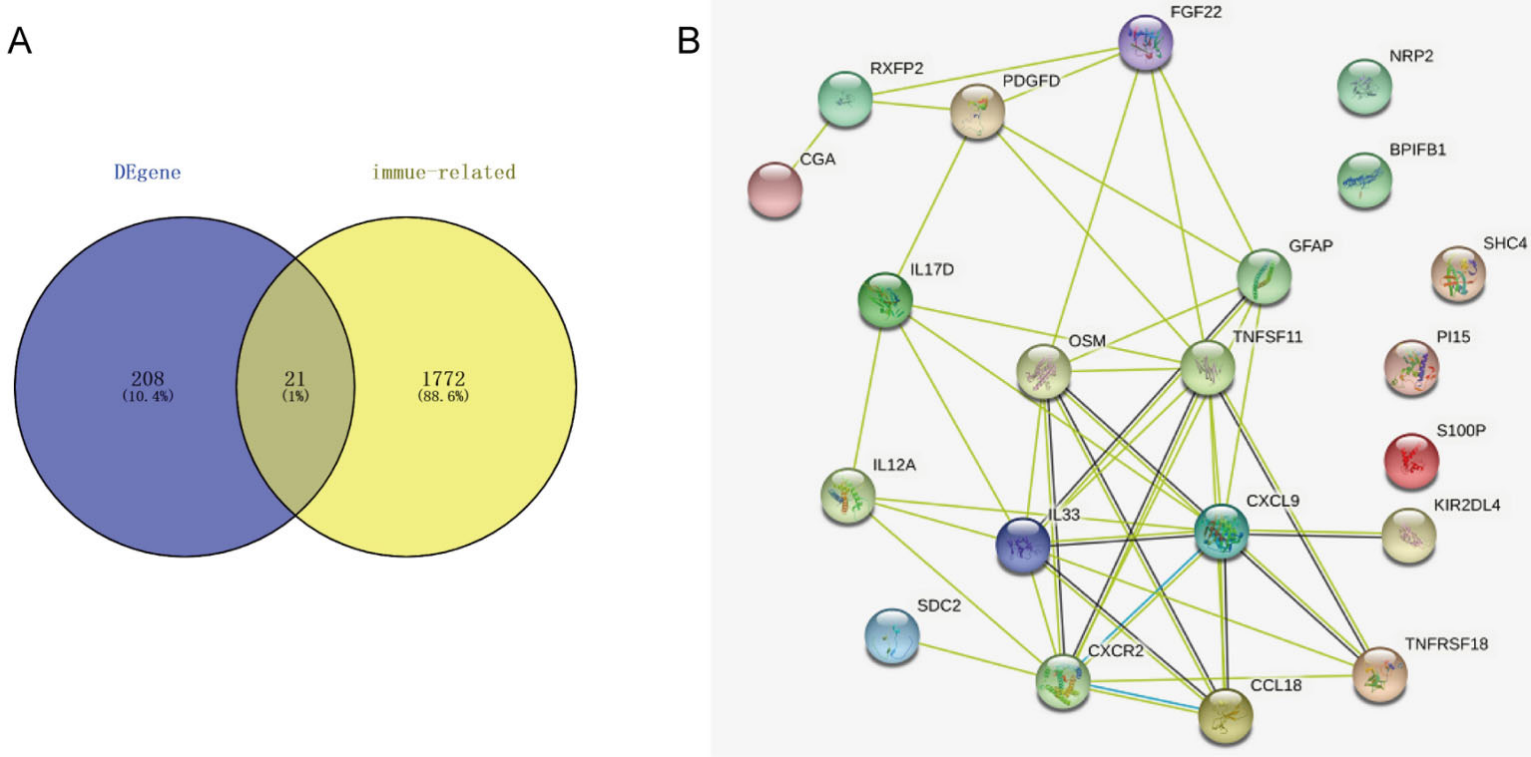
Identification of immune-related differentially expressed genes (DEGs). **A**, Venn diagrams show the intersection of DEGs and immune genes, identifying immune-related DEGs. **B**, Protein-protein interaction (PPI) network displays the interactions among immune-related DEGs.

We acquired 1793 immune-related genes from the Immport database. Subsequently, immune-related genes and DEGs were overlapped to obtain 21 immune-related DEGs (Figure 3A), including S100 calcium binding protein P (*S100P*), syndecan 2 (*SDC2*), C-C motif chemokine ligand 18 (*CCL18*), C-X-C motif chemokine ligand 9 (*CXCL9*), TNF superfamily member 11 (*TNFSF11*), relaxin family peptide receptor 2 (*RXFP2*), killer cell immunoglobulin like receptor, two Ig domains and long cytoplasmic tail 4 (*KIR2DL4*), interleukin 33 (*IL33*), oncostatin M (*OSM*), interleukin 17D (*IL17D*), BPI fold containing family B member 1 (*BPIFB1*), interleukin 12A (*IL12A*), glycoprotein hormones, alpha polypeptide (*CGA*), glial fibrillary acidic protein (*GFAP*), SHC adaptor protein 4 (*SHC4*), peptidase inhibitor 15 (*PI15*), fibroblast growth factor 22 (*FGF22*), C-X-C motif chemokine receptor 2 (*CXCR2*), platelet derived growth factor D (*PDGFD*), neuropilin 2 (*NRP2*), and TNF receptor superfamily member 18 (*TNFRSF18*).

To investigate the interactions among the 21 immune-related DEGs, the PPI network was performed. The result revealed that 16 immune-related DEGs had mutual relationships (Figure 3B). The interaction between them may promote the occurrence and development of STEMI.

### Correlation analysis between immune-related DEGs and immune cells

**Figure 4:**
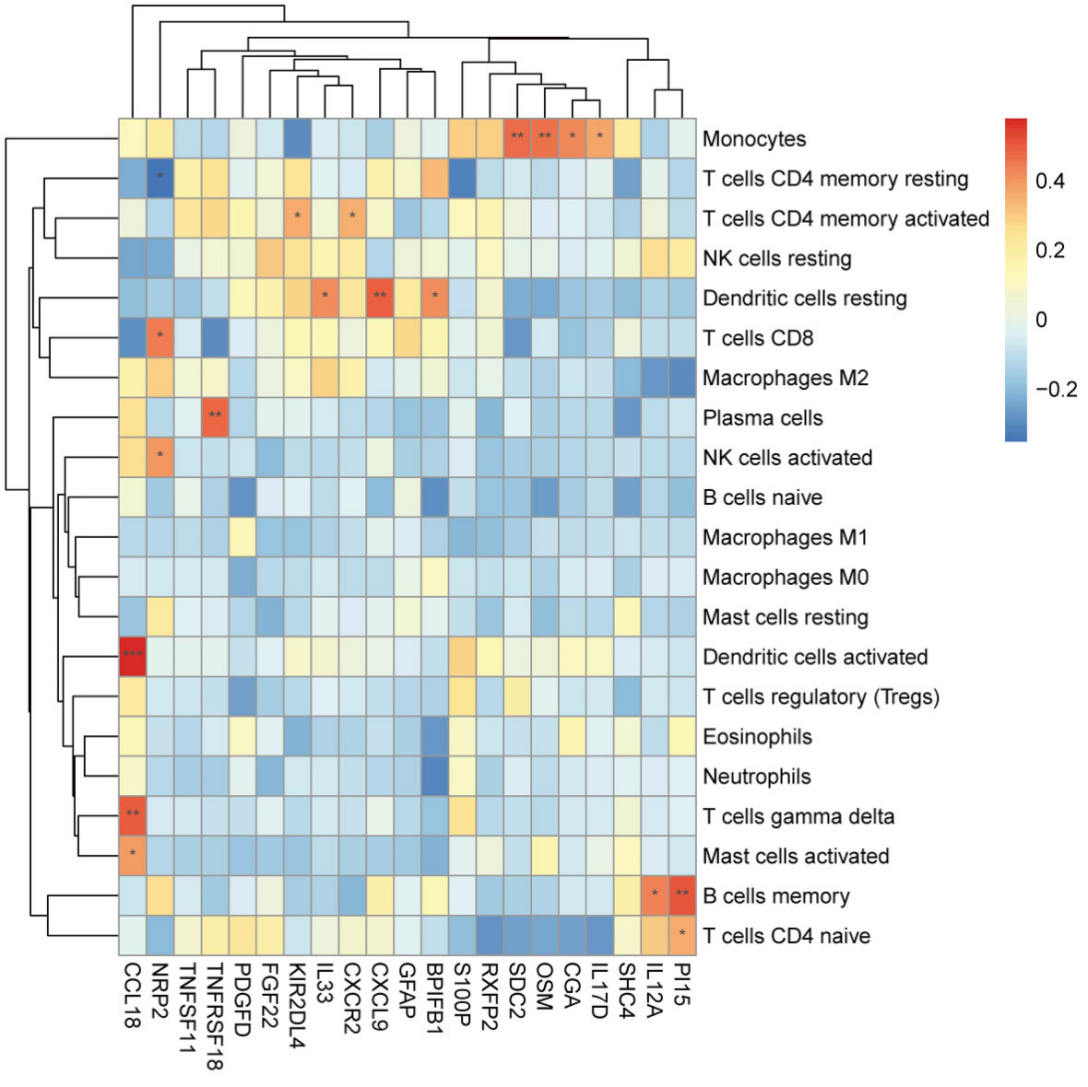
Correlation analysis between immune-related differentially expressed genes (DEGs) and immune cells. *P<0.05, **P<0.01, ***P<0.001 (Pearson correlation).

Correlation analysis showed that *CCL18* had a significant and positive correlation with activated dendritic cells, T cells gamma delta, and activated mast cells; *NRP2* was significantly negatively related to resting CD4 memory T cells, and significantly positively associated with activated CD8 T cells and NK cells; *TNFRSF18* was significantly positively connected with plasma cells; *KIR2DL4* and *CXCR2* were significantly positively relevant to activated CD4 memory T cells; *SDC2*, *OSM*, *CGA*, and *IL17D* were significantly positively related to monocytes; *IL12A* was significantly positively connected with memory B cells (Figure 4).

### Analysis of key genes using RT-PCR

**Figure 5:**
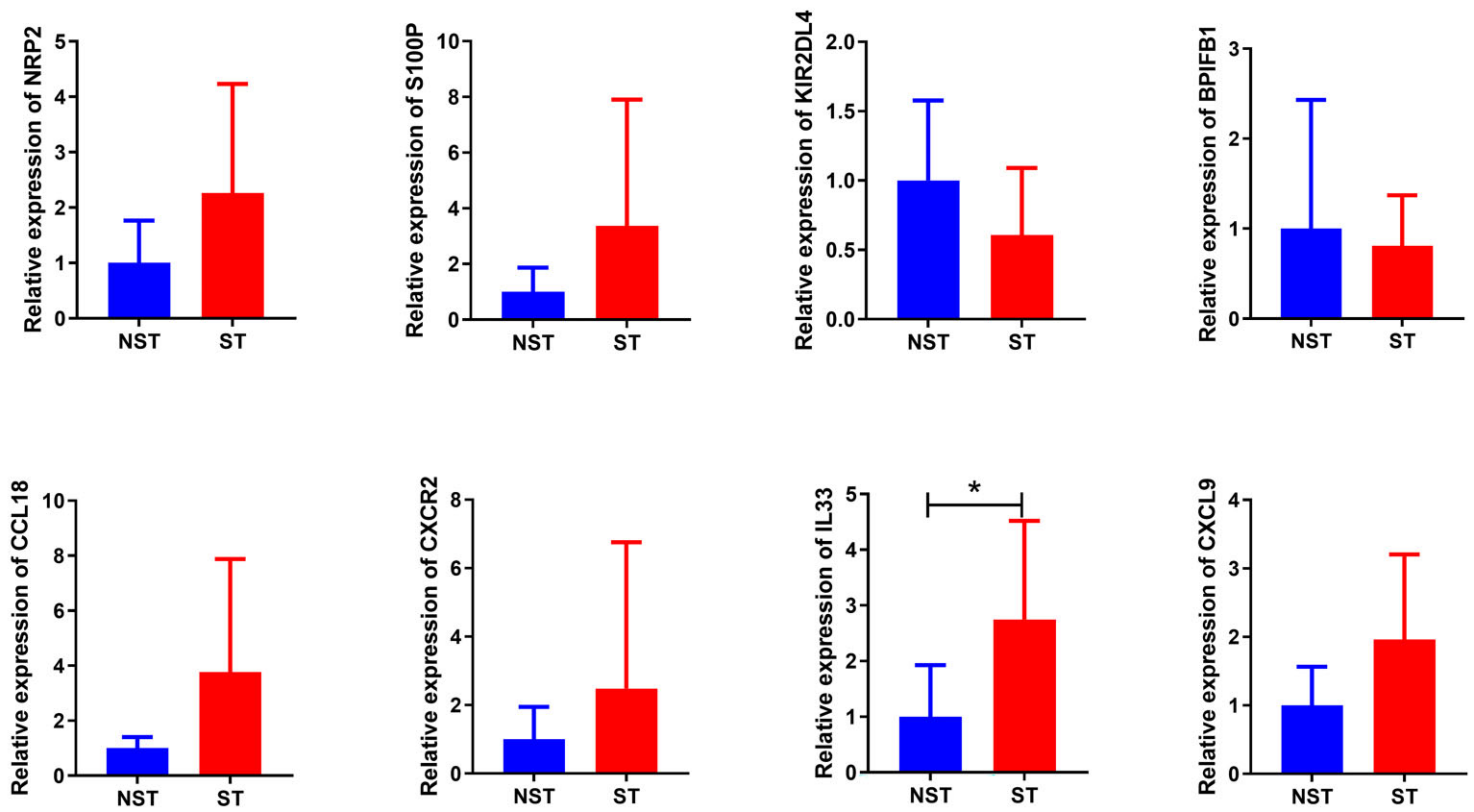
Key gene validation. Expression of immune-related differentially expressed genes (DEGs) (*NRP2*, *S100P*, *KIR2DL4*, *BPIFB1*, *CCL18*, *CXCR2*, *IL33*, and *CXCL9*) examined in ST-segment elevation myocardial infarction (ST) (n=9) and non-ST elevation myocardial infarction (NST) (n=6) samples using real-time PCR. Data are reported as means and SD. *P<0.05 (*t*-test).

RT-PCR results showed that the expression of *NRP2*, *S100P*, *CCL18*, *CXCR2*, *IL33*, and *CXCL9* had an up-regulated tendency in STEMI group compared with NSTEMI group, and the expression of *IL33* was significant (Figure 5) (see Supplementary Table S2 for RT-PCR validation data). Conversely, the expression of *KIR2DL4* and *BPIFB1* showed a down-regulation trend.

## Discussion

AMI remains one of the leading causes of mortality worldwide (4). Early and accurate diagnosis can reduce mortality and improve prognosis (13). The intricate involvement of various immune cells in the pathology of AMI at different stages needs a deeper understanding (9). We investigated the differences in immune cell infiltration in STEMI and NSTEMI and the correlation between immune cells and key genes, which may aid in the exploration of AMI treatments.

The infiltration of immune cells exhibited significant differences between STEMI and NSTEMI, including increases in activated dendritic cells and CD4 memory T cells, and reductions in resting mast cells and CD8 T cells in STEMI compared to NSTEMI. These findings align with previous observations of decreased resting mast cells and CD8 T cells infiltration in STEMI (14,15). The modulation of mast cell activity is associated with altered plaque stability, and plays an important role in AMI (16-18). Additionally, the significant infiltration of activated dendritic cells in STEMI may potentially contribute to the severity of the infarction by facilitating more extensive inflammation and tissue damage (19). Correlation analysis further unveiled significant associations among specific immune cell subpopulations, which may influence the progression of the myocardial injury and the subsequent healing process (20). Overall, our results demonstrated the different immune signatures of STEMI and NSTEMI.

A total of 229 DEGs were identified between STEMI and NSTEMI. GO analysis showed that these DEGs were mainly involved in the positive regulation of cell differentiation and cell proliferation, extracellular region and calcium ion binding, which were closely associated with AMI (21). Furthermore, KEGG results revealed that cytokine-cytokine receptor interaction and drug metabolism-cytochrome P450 were the only two significantly enriched signaling pathways. The drug metabolism-cytochrome P450, an important signal transduction pathway in cells, is essential for cell survival (22). Cytokines are extracellular molecules that transmit signals between cells, which are widespread in cell differentiation and inflammatory responses (23). These results suggested potential differences in inflammatory regulation and drug metabolism processes in NSTEMI and STEMI patients.

Subsequently, 21 immune-related DEGs were identified, the majority of which showed positive correlations with immune cells. For example, *CCL18* is produced by dendritic cells in the germinal centers of secondary lymphoid organs (24), and was significantly positively correlated with activated dendritic cells, gamma delta T cells, and activated mast cells. *NRP2*, which is intricately involved in vascular atherosclerosis, was correlated positively with CD8 T cells. CD8 T cells are known for their dual role in cardiac remodeling (25,26). Moreover, PPI analysis revealed mutual interactions among 16 of these genes, possibly providing a framework for understanding the complex regulatory mechanisms of immune responses in AMI.

Previous research showed that interleukin (IL)-33 can attenuate the level of inflammation and myocardial apoptosis after AMI, and has cardioprotective effects on various cardiovascular diseases (27). CXCR2 can reduce myocardial injury following myocardial ischemia-reperfusion injury (28,29). CXCR2 may play a critical cardioprotective effect in AMI by upregulation of cardiac adhesion molecules (30). Serum CXCL9 levels were significantly elevated in AMI patients (31). *CCL18* is expressed by monocytes/macrophages and dendritic cells (32). Median serum concentrations of CCL18/PARC are significantly higher in cardiovascular patients (33). Cai et al. (34) have proven that the expression of S100P is related to acute coronary syndrome in rat models, and the level of S100P in STEMI patients is higher than that in NSTEMI patients. NRP2 supports lymphangiogenesis and neovascularization, and its inhibition might be a promising therapeutic approach for occlusive vascular diseases. However, given its role in promoting lymphangiogenesis, inhibition of NRP2 may promote plaque growth in advanced atherosclerosis, suggesting a complex role in AMI. Most of the studies on KIR2DL4 and BPIFB1 are tumor-oriented, and the expression of *KIR2DL4* and *BPIFB1* in AMI samples is down-regulated, which may be helpful for AMI research. Collectively, these immune-related DEGs demonstrated a significant correlation with immune cells, and the expression patterns of these genes showed differences between NSTEMI and STEMI patients, suggesting their potential as targets for immunotherapy. However, the observed differences in expression were not statistically significant, which may be due to the limited sample size, underscoring the need for further validation in a larger cohort.

### Conclusion

In conclusion, our study explored the abundance and differences of STEMI and NSTEMI-specific immune cell subsets in AMI, identified key immune-related genes, and elucidated their relationships with immune cell infiltration as well as the expression of key genes. This comprehensive analysis may offer valuable insights into the immune mechanisms at play in AMI.

## Supplementary Material

Click here to view [zip].
