# Supplementary material for: Analysis of the differences in immune-related genes and immune cell subtypes in acute myocardial infarction
Source: Braz J Med Biol Res. 2024 Oct 14;57:e14345. doi: 10.1590/1414-431X2024e14345 (PMC11484353; doi:10.1590/1414-431X2024e14345)
Supplement: Supplementary file 1 [file 1414-431X-bjmbr-57-e14345-suppl.zip › 14345_Supplementary Table S1.pdf]

Table S1. Clinical information registry.

Number	Groups	Age (years)	Gender	Height (cm)	Weight (kg)	BMI	HDL (mmol/L)	LDL (mmol/L)	TC (mmol/L)	cTnI (ng/mL)	Alcohol history	Smoking history	Family history
1	STEMI	52	Male	170	60	20.76	1.1	2.86	9.12	5.8623	Yes	No	No
2	STEMI	71	Female	158	55	22.03	1.49	2.19	0.59	>40	No	No	No
3	STEMI	58	Male	175	61	19.92	1	2.22	0.67	13.63	No	Yes	No
4	STEMI	72	Male	175	70	22.9	1.28	3.22	1.27	0.14	No	Yes	No
5	STEMI	49	Male	168	70	24.8	1.13	2.81	1.14	1.246	No	No	Hypertension
6	STEMI	58	Male	168	69	24.4	0.95	3.24	0.94	>40	No	No	No
7	STEMI	73	Female	160	60	23.44	1.63	3.74	1.29	36.415	No	No	No
8	STEMI	79	Female	153	62	26.5	1.34	2.73	1.28	>40	No	No	No
9	STEMI	47	Male	165	80	29.4	1.34	5.05	0.96	>40	No	Yes	No
10	NSTEMI	68	Male	160	55	21.5	1.35	1.32	1.27	9.2904	Yes	Yes	No
11	NSTEMI	70	Male	175	68	22.2	1.59	3.07	0.59	2.1126	Yes	Yes	No
12	NSTEMI	73	Female	155	60	25	1.16	1.72	1.29	0.85	No	No	No
13	NSTEMI	49	Male	173	68	22.7	1.05	2.44	4.41	>40	Yes	Yes	No
14	NSTEMI	39	Male	175	70	22.9	1.02	3.24	2.2	3.1	No	Yes	No
15	NSTEMI	66	Male	170	68	23.53	2.20	2.25	0.6	0.3	No	Yes	–

BMI: Body mass index; HDL: high-density lipoprotein; LDL: low-density lipoprotein; TC: total cholesterol; cTnI: cardiac troponin I; STEMI: ST-segment elevation myocardial infarction; NSTEMI: non-ST elevation myocardial infarction.
